# Large-scale microlens arrays on flexible substrate with improved numerical aperture for curved integral imaging 3D display

**DOI:** 10.1038/s41598-020-68620-z

**Published:** 2020-07-16

**Authors:** Wenwen Wang, Guixiong Chen, Yalian Weng, Xuyang Weng, Xiongtu Zhou, Chaoxing Wu, Tailiang Guo, Qun Yan, Zhixian Lin, Yongai Zhang

**Affiliations:** 1 College of Physics and Information Engineering, Fuzhou University, Fuzhou, 350116 Fujian People’s Republic of China; 2 Fujian Science and Technology Innovation Laboratory for Optoelectronic Information of China, Fuzhou, 350116 Fujian People’s Republic of China

**Keywords:** Displays, Optoelectronic devices and components

## Abstract

Curved integral imaging 3D display could provide enhanced 3D sense of immersion and wider viewing angle, and is gaining increasing interest among discerning users. In this work, large scale microlens arrays (MLAs) on flexible PMMA substrate were achieved based on screen printing method. Meanwhile, an inverted reflowing configuration as well as optimization of UV resin’s viscosity and substrate’s surface wettability were implemented to improved the numerical aperture (NA) of microlenses. The results showed that the NA values of MLAs could be increased effectively by adopting inverted reflowing manner with appropriate reflowing time. With decreasing the substrate’s wettability, the NA values could be increased from 0.036 to 0.096, when the UV resin contact angles increased from 60.1° to 88.7°. For demonstration, the fabricated MLAs was combined to a curved 2D monitor to realize a 31-inch curved integral imaging 3D display system, exhibiting wider viewing angle than flat integral imaging 3D display system.

## Introduction

Display technology will develop in the direction of more natural vision and more user-friendly. In the past two decades, considerable attention has been paid to extend the classical two-dimensional (2D) displays into their three-dimensional (3D) counterparts because of its stronger sense of reality^1–4^. Another important research stream is flexible display, which has thin, lightweight and non-breakable characteristics. Displays with flexible form factors enable the fabrication of displays on curvilinear surfaces and allow their shapes to be transformed, providing potential applications to mobile, wearable and vehicle display. Therefore, flexible 3D displays become a major technological and application goal in the field of next-generation displays.

Integral imaging is an autostereoscopic and multiscopic 3D display technology that uses double micro-lens arrays (MLAs) to capture and reproduce a light field of the target based on reversibility principle of light rays^5^. It is regarded as a promising approach to realize the 3D display system due to its typical characteristics, such as glasses free, full parallax, quasi-continuous view points, eliminating visual fatigue and real 3D display. In particular, it produces real concentrations of light to optically produce 3D images that are observed without decoupling the convergence and the accommodation, avoiding the detrimental effects of the convergence-accommodation conflict. Thus, there has been substantially increasing interest in researching and implementing effective technologies for the capture, processing, and display of 3D images^6–8^. It is also inferred that flexible integral imaging 3D displays could be achieved via the combination of flexible 2D display panel and flexible MLAs^9–11^.

MLAs have been widely used as key components in lots of optical systems^12–14^, such as integral imaging^15,16^, optical communications^17^, digital display^18^, detection^19^ and far field imaging^20^. In particular, MLAs with good converging performance, great uniformity of focusing and good repeatability of geometry parameters in large-scale is of great important for the prototype and industrialization of integral image 3D display. A variety of fabrication strategies have been developed including screen printing^21^, gray-scale photolithography^22^, photo-resist reflow method^23–25^, two-photon polymerization^26,27^, nano-imprinting^28^ and ink-jet printing^29,30^, etc. Among the above methods, screen printing has been proved to be an efficient fabrication method of large-scale and high-performance MLAs for integral imaging 3D display^21^. Furthermore, it might be feasible to achieve flexible MLAs by transferring UV resin onto flexible substrates. Although screen printing is particularly suitable for the fabrication of MLAs with large-scale, it still faces many technical difficulties for the fabrication of MLAs with large diameter of individual micro-lens, which is normally required in the large-scale integral imaging 3D display system to adapt to the pixel of display panels. For instance, the numerical aperture (NA) will influence distinctly the light collection efficiency and converging performance, low NA will lead to more crosstalks and smaller depth of field in the integral imaging 3D display. However, the NA of MLAs fabricated by screen printing is relative low since the micro-lenses are limited in height (20–30 μm) and large in diameter (more than 1000 μm)^31^. What’s more, screen printing technology takes advantage of surface tension of UV resin to form spherically shaped microlenses with smooth profiles. This surface-tension-assisted manner could be exploited to further control the profiles of microlenses^32^.

On the other hand, curved displays are now quickly overtaking flat monitors among discerning users, due to a number of benefits unique to their design, including enhanced sense of immersion, wider field of view and reduced eyes strain^6,33–35^. The realization of curved integral imaging 3D display not only have great application potentials, but also provide referential values to flexible 3D displays when they are under deformation^36,37^. However, besides the fabrication issue, the design of FMLAs is also important to achieve high performance reconstruction image, since the flexible MLAs and the 2D display panel are located in different curved surfaces.

This work focuses on the augmentation of NA of flexible MLAs with the diagonal size of 31 inches that were fabricated on flexible substrate using screen printing. For demonstration, the flexible MLAs were used for curved integral imaging 3D display system, exhibiting high light collection efficiency and good reconstruction performance.

## Results and discussion

### Increased NA of microlenses via inverted reflowing manner

During the screen printing processes, micro-cylinder arrays (MCAs) are first formed on the substrates when the UV resin is extrude from the open mesh by the scraper. The micro-cylinders will then spread out and form spherically shaped microlenses under the synergistic effects of gravity and surface tension of UV resin. According to a reflowing model^38^, the edges of the UV resin is inclined to flow out under the gravity, while the surface tension tries to minimize the surface area. In this work, in order to take advantage of the gravity effect, an inverted reflowing configuration was implemented, that is to say the substrate was flipped with MCAs face down to reflow until spherically shaped MLAs were formed, followed by solidifying under UV light (365 nm).

**Figure 1:**
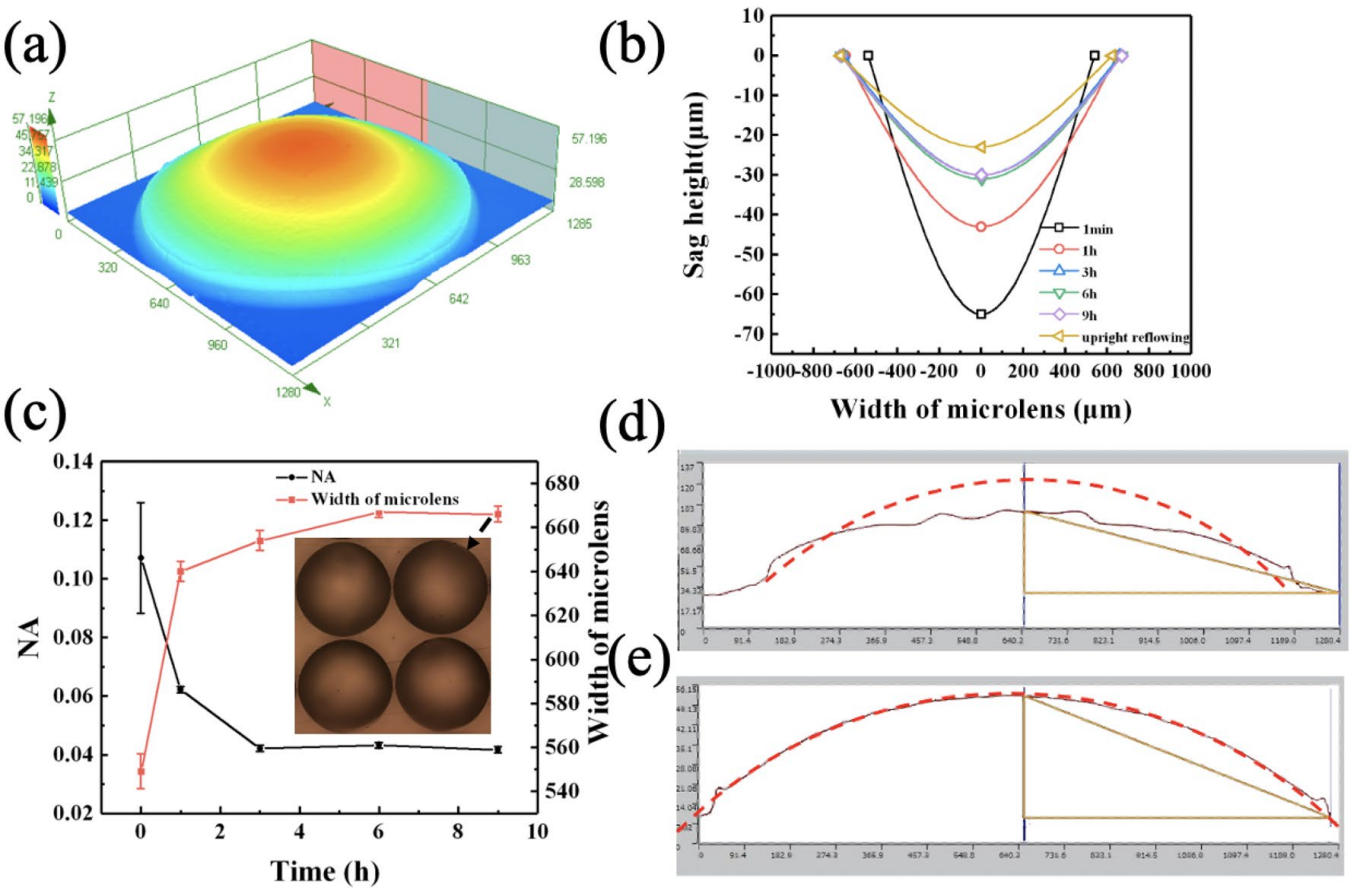
Morphology of MLAs with inverted reflowing manner. (**a**) 3D perspective images of a typical solidified microlens with inverted reflowing time of 1 h. (**b**) The profiles of MLAs with respect to reflowing time. (**c**) The evolution of NA and radius of MLAs with the reflowing time. The height profiles (red solid line) taken along the equatorial plane of a typical microlens and fitting of an ideal surface to the lens profiles (Red dash line) with inverted reflowing time of 1 min (**d**) and 1 h (**e**).

The morphology of the MLAs were characterized using laser three-dimensional (3D) microscope. Besides two- dimensional (2D) image, the laser 3D microscope can scan layer by layer in the Z-axis direction and reconstruct 3D geometric morphology of objects. Figure 1a illustrated a 3D perspective image of a typical solidified microlens with inverted reflowing time of 1 h, showing spherically shaped profile. With 3D perspective images, the profiles of MLAs with respect to reflowing time were investigated. Figure 1b depicts the profiles of MLAs with inverted reflowing time of 1 min, 1 h, 3 h, 6 h and 9 h, respectively. As a reference, the profiles of MLA with conventional reflowing manner (upright reflowing) was also illustrated in Fig. 1b. When the printed MCAs were flipped within short time (e.g., 1 min), the height of MLAs at the vertex increased significantly and the diameter of MLAs shrinked obviously, indicating that the gravity played an important role in the formation of lens profile at the early stage. Afterwards, the height decreased and the diameter recovered gradually due to the surface tension. The dynamic equilibrium reached after 3 h, and the profile of MLAs showed little change. The MLAs fabricated with inverted reflowing manner exhibited larger height than that with upright reflowing manner.

The evolution of the profile and focus performance of MLAs with reflowing time (from 1 min to 9 h) was further described by the values of NA and focus length of microlens (f) according to formula (1) and (2), where n_0_ = 1 was the surrounding background refractive index, $\theta$ was half of the focusing angle which can be deduced by focal length and radius, r was the radius, h was the height of microlens at the vertex, n = 1.476 was the refractive index of solidified UV resin.

1 $$NA = n_{0} \sin \theta$$

2 $${\text{f}} = \frac{{{\text{r}}^{2} + {\text{h}}^{2} }}{{2{\text{h}}\left( {{\text{n}} - 1} \right)}}$$

The relationships of NA and radius (r) of MLAs with the reflowing time (t) were showed in Fig. 1c, presenting similar variation tendency to that of microlens height. It was noted that, although the calculated NA of MLAs with inverted reflowing time of 1 min was much larger than those with longer reflowing time, spherical surfaces were not yet formed. Figure 1d, e illustrated the height profiles (red solid line) taken along the equatorial plane of a typical micro-lens for MLAs with inverted reflowing time of 1 min and 1 h, respectively. The fitting of an ideal surface to the lens profiles (Red dash line) was also provided for reference. It was evident that the shape of MLAs was strongly affected by the reflowing time, a severe sag with serrated surface appeared on the center of the microlens. After 1 h of reflowing, the profile of microlens exhibited a very small profile deviation with the ideal spherical surface.The value of NA of microlens with inverted reflowing time of 1 h was around 0.063,which was much higher than that with upright reflowing manner (0.036).

### Effects of viscosity of UV resin on the morphology of MLAs

**Figure 2:**
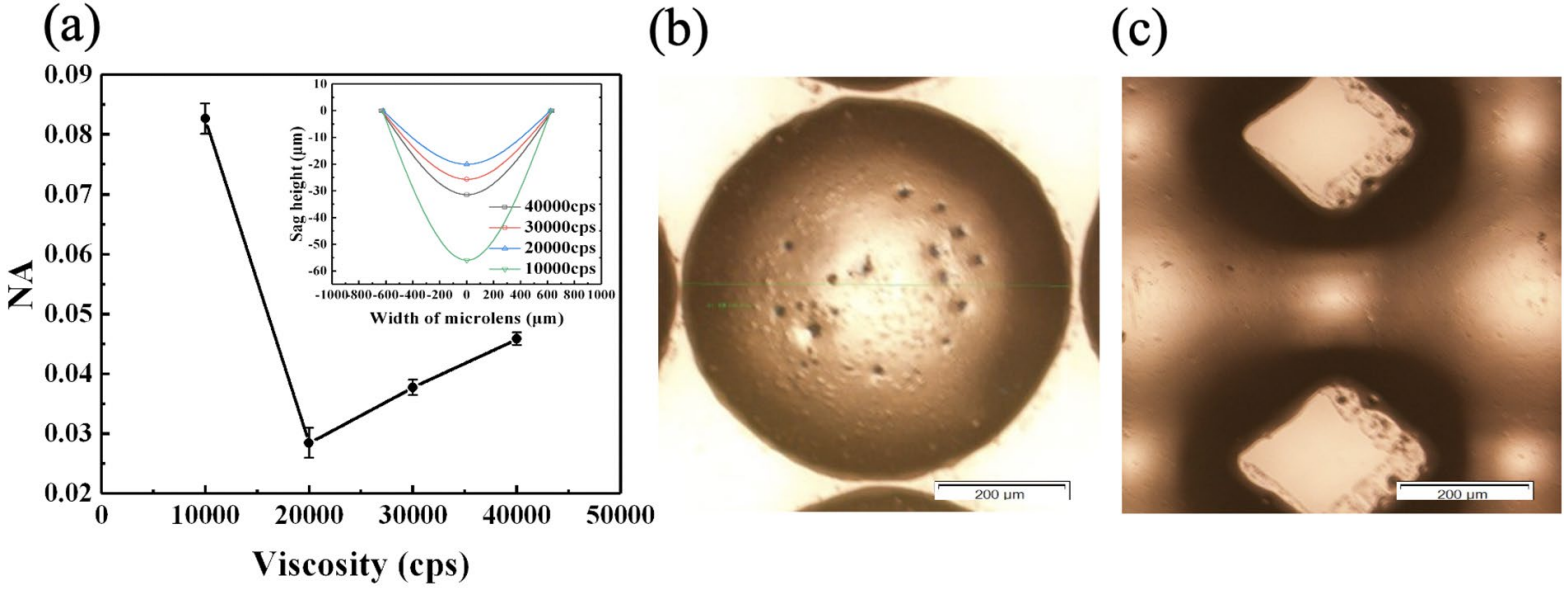
(**a**) The relationship between the NA value and the viscosity of UV resin, the inset presents the profiles of MLAs with respect to viscosity of UV resin. (**b**) Microscope image of a typical microlen fabricated using UV resin with viscosity of around 40,000 cps, showing the existence of bubbles on the lens surface. (**c**) Microscope image of MLAs fabricated using UV resin with viscosity of around 10,000 cps, showing linkages between adjacent microlens.

In order to further understand the spreading behaviours of UV resins under the inverted reflowing configuration, the effects of viscosity of UV resin on the morphology of MLAs were also investigated. The viscosity of UV resin was adjusted by altering the volume ratios of UV resin with high viscosity of around 50,000 cps and low viscosity of around 4000 cps. UV resins with viscosities of around 40,000 cps, 30,000 cps, 20,000 cps and 10,000 cps were used respectively. The relationships between NA and viscosity of UV resin were described in Fig. 2a, where the profiles were also embodied in the inset. It was generally accepted that UV resin with higher viscosity would decrease their spreading. Therefore,the NA of MLAs increased by increasing the viscosity of UV resin from 20,000 to 40,000 cps. However, when the UV resin was too viscous, air bubbles were hard to be eliminated, as shown in Fig. 2b. These bubbles would cause light scattering during imaging process. Unexpectedly, the MLAs fabricated using UV resins with viscosities of around 10,000 cps showed the largest NA, which might be attributed to the easier flowability under the gravity effect. However, this easier flowability would cause linkage problems between adjacent microlens, as shown in Fig. 2c.

### Effects of surface property of substrate on the morphology of MLAs

**Figure 3:**
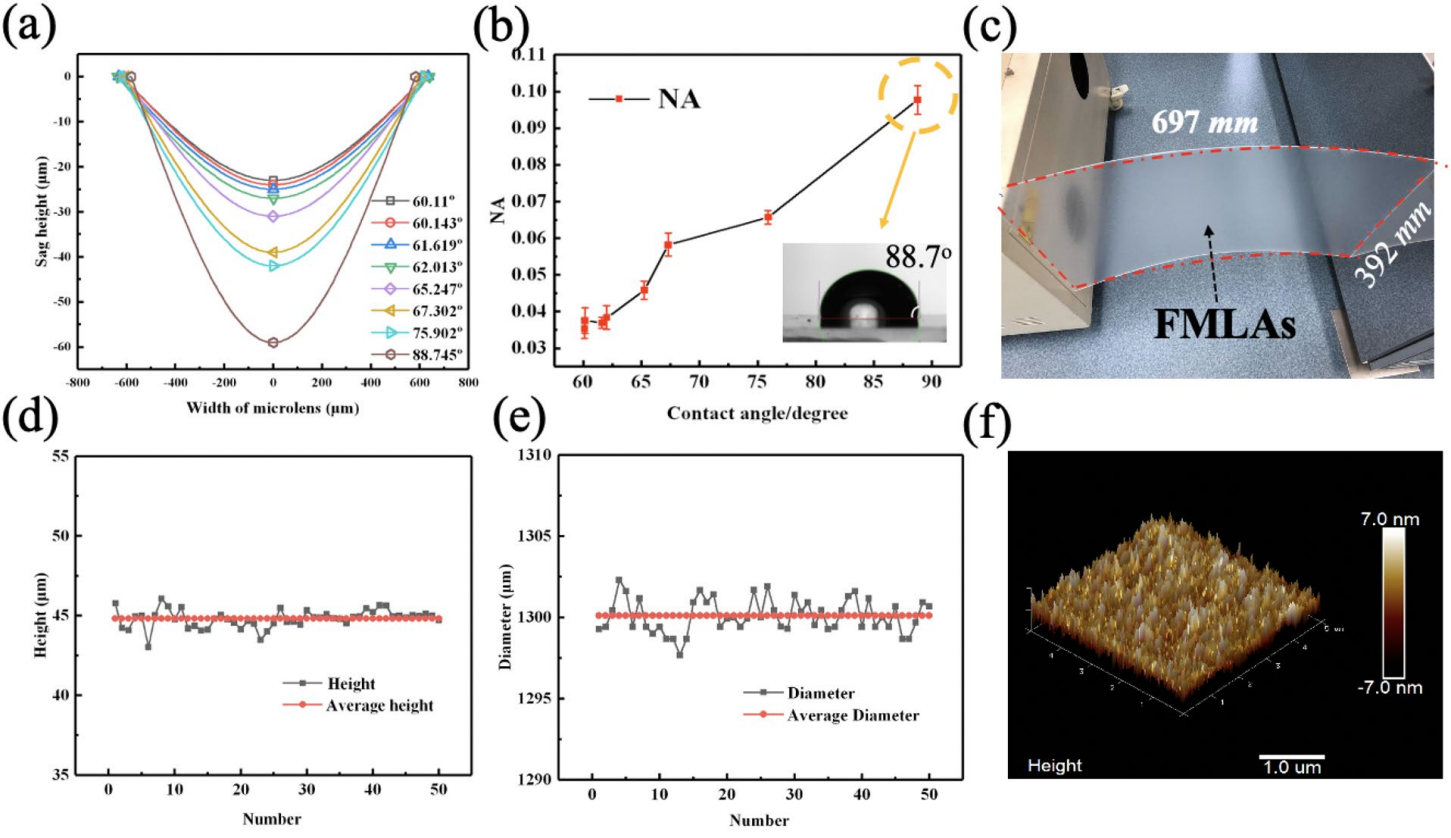
(**a**) The lens profile for MLAs fabricated on PMMA substrates with various contact angles of UV resin. (**b**) The NA of microlenses as a function of surface contact angle, the inset shows a typical optical micrograph of a drop of UV resin on PMMA substrate showing surface contact angle of 88.7°. (**c**) The photo of a 31-inch MLAs fabricated on PMMA substrate with TMCS treatment of 55 min and with inverted reflowing time of 1 h. The uniformity information in terms of the height (**d**) and diameter (**e**) information of 50 randomly selected micro-lenses from the MLAs shown in Fig. 4c. (**f**) A typical AFM image of the surface of micro-lens over a 5 μm × 5 μm area, showing excellent surface quality.

The surface tension of UV resin was tailored via regulating the surface wetting conditions of the PMMA substrates to further improve the NA of MLAs. Specifically, the PMMA substrates were hydrophobically treated using TMCS vapor for various time, and the static UV resin (30,000 cps) contact angles on PMMA surfaces were measured to determined the wettability. The UV resin contact angles on PMMA surfaces with TMCS modification time of 0 min (without TMCS treatment), 5 min, 10 min, 20 min, 35 min and 55 min were 60.1 ± 0.5°, 62.0 ± 1.2°, 65.2 ± 2.5°, 67.3 ± 3.5°, 75.9 ± 3.2° and 88.7 ± 0.2°, respectively, showing decreasing wettability with respect to the TMCS modification time. Figure 3a demonstrated the profiles of MLAs fabricated on PMMA surfaces with different wettability. It was obvious that the height of microlenses increased with decreasing wettability. The height and sphericity of a lens is determined by the force balance between surface tension and gravity. If the substrate was less wettable, the interaction force between the UV resin and the PMMA substrate was larger, thus impeding the spreading of UV resin. Figure 3b plotted the curve of NA versus contact angle, showing that the NA values increased proportionally to the substrate’s contact angle. The NA could be increased from 0.036 to 0.096 after TMCS modification (55 min). Although the value of NA (0.096) obtained using screen printing was still relative low compared to that fabricated using other methods, it provided a simple and potential fabrication strategy of large-scale flexible MLAs for integral imaging 3D display. The NA of MLAs by screen printing could be further improved by adding larger amounts of UV resin in each microlens, e.g. using screen with larger thickness.

Flexible MLAs with size of 697 mm (length) × 392 mm (width) were achieved by printing UV resin (30,000 cps) on PMMA substrate with UV resin contact angle of around 88.7°, followed by inverted reflowing for 1 h and UV solidification, as demonstrated in Fig. 3c. By using flexible PMMA substrate, the MLAs could be bent to some extent, in order to adapt curved screens with different curvatures. The uniformity in terms of the diameter and height was examined, as shown in Fig. 3d, e. The diameter and height of the microlenses were 1300 ± 2.3 μm and 44.81 ± 1.4 μm, respectively, showing good uniformity. The roughness of the microlen surface was also verified by atomic force microscope (AFM), Fig. 3f showed a typical surface topography of a 5 × 5μm^2^ scanning area. Dozens of microlenses were selected for AFM observation, from which excellent surface quality with average Ra of 1.6 ± 0.1 nm could be found. The smooth surface was expected to have good image reconstruction performance in integral imaging 3D display system.

### Characterization of optical parameters

**Figure 4:**
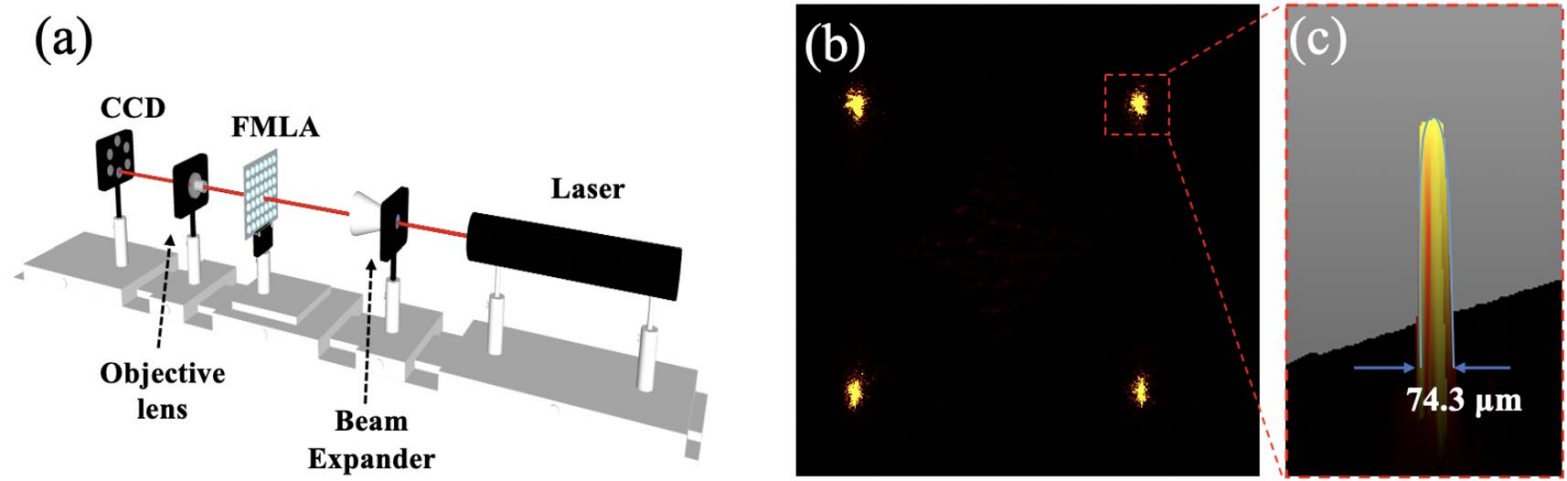
The focusing performances of the MLAs. (**a**) Schematic of experimental setup for examining the optical properties of the fabricated MLAs. (**b**) the 2D light intensity distribution with the sharpest focus spots. (**c**) The corresponding 3D light intensity distribution of a randomly selected focused spots.

To verify the focusing performance of the MLAs, an experimental setup was built, as schematically shown in Fig. 4a. A parallel laser beam with wavelength of 632.8 nm was expanded and was normally incident on the MLAs. The exiting light was focused on a objective lens, and then a magnified image was recorded by CCD camera for analysis. Since the NA of MLAs was relatively low, the radius of Airy disc was large compared to the pixel size of the CCD camera (6.45 × 6.45 μm). Instead of comparing with the Airy disc, the focusing performance of microlenses were evaluated qualitatively by the area ratios of the focus spot to the microlens. Figure 4b showed a 2D light intensity distribution image, where the area of focus spots and the distance between focus spots were magnified with the same proportion. The center distance between two neighboring microlenses was 1362 μm, which was taken as reference for calculating the effective diameters of the focal points. Figure 4c display a magnification of 3D light intensity distribution, whose outline was found to meet approximately Gaussian distribution. Thus, the effective diameters of the focal points were taken as 74.3 μm when the light intensity was A = A_0_/e, where A_0_ was the maximum intensity of the focus spot and e ~ 2.72 was the Euler number. The area of focal points was calculated and accounted for 0.33% with respect to that of micro-lens. Although the focus spots were not rotationally symmetric, the light spots were sharp, indicating good converging performance. The asymmetric shapes might be attributed to the stray light caused by the reflected light from the substrates or the scattered light due to bubbles in the MLAs.

### Construction and performances of curved integral imaging system

**Figure 5:**
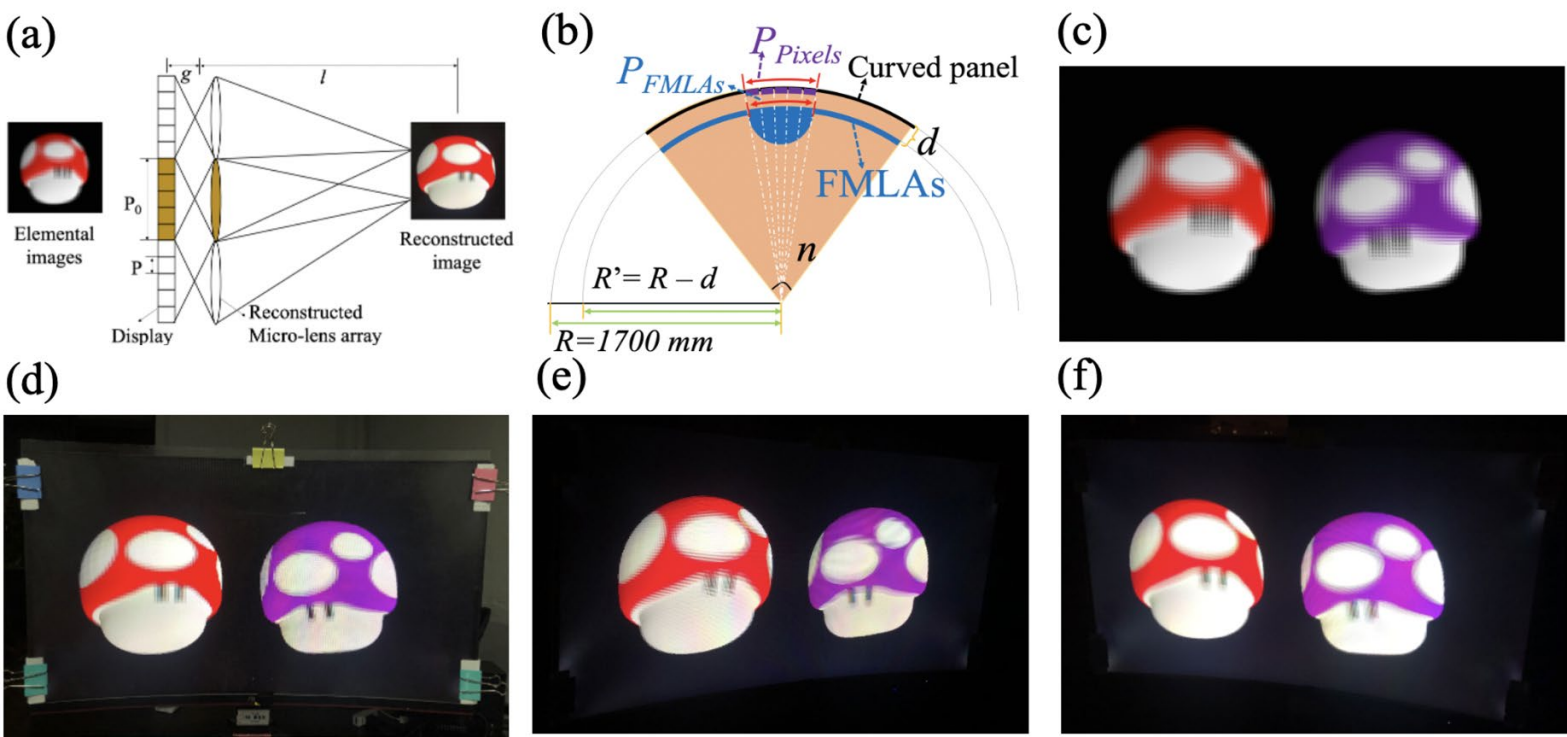
(**a**) Scheme of reconstruction stage of integral imaging, a microlens covered 5 × 5 pixels. (**b**) Design scheme of microlens’s pitch for the curved integral imaging 3D display system. (**c**) An elemental image array captured using computer graphics techniques. (**d**) The photo of constructed 31-inch integral imaging 3D display system with a floating 3D reconstruction image viewed from the normal direction. (**e**) A photo of 3D reconstruction image viewed from the left 45° direction. (**f**) A photo of 3D reconstruction image viewed from the right 45° direction.

In the integral imaging process, every microlens in the MLAs captured a 2D picture of the 3D scene from a different perspective, these 2D pictures could be grouped to form elemental image arrays, in which every individual perspective image contained the angular information corresponding to rays passing through the vertex of the corresponding microlens. The elemental image arrays were then displayed on a 2D panel, which was put behind a virtual MLA. Floating 3D images were then produced in front of the 2D monitor according to the reversibility of optical path, as shemtaically illustrated in Fig. 5a.

To confirm the imaging performance of fabricated flexible MLAs, an implementation of integral imaging 3D display system using a curved monitor with pixel pitch of 272.4 μm and radius of 1700 mm was demonstrated. For the integral imaging 3D display, the observer views a single pixel through each microlens. Thus, the display resolution was determined by the pitch of the MLA. In our experiment, considering the trade-off between the angular continuously smooth and and the spatial resolution, the pitch of MLAs was designed such that each microlens covered 5 × 5 pixels on the 2D monitor. Thus, the resolution of integral imaging 3D display system should be 512 × 288 for 2D monitor with resolution of 2560 × 1440. For flat integral imaging 3D display system, the pitch of MLAs should be 1362 μm × 1362 μm in the horizontal and vertical directions. However, if the MLAs was bent to a certain curvature, the pitch of MLAs in the horizontal direction should be redesigned to eliminate the accumulative error, since the 2D curved monitor and the MLAs were located in two concentric circle with different radii of curvature, as shemtaically depcited in Fig. 5b. Generally, the MLA should be set such that its focal plane coincides with the position of the panel’s pixels, in order to allow the reconstruction image to be as clear as possible. In order to compensate the displacement between MLAs and 2D monitor, the pitch of MLAs in the horizontal direction could then be calculated according to formula (3),

3 $$\frac{{{\text{P}}_{{{\text{pixels}}}} }}{{{\text{P}}_{{{\text{FMLA}}}} }} = \frac{{\text{R}}}{{{\text{R}} - {\text{d}}}}$$

where P_pixels_ was the lenth of 5 pixels in the 2D curved panel, P_FMLA_ was the pitch of flexible MLAs in the horizontal direction, R = 1700 mm was the radius of curvature of the 2D curved panel, d was the distance between MLAs and 2D curved panel, which was equal to the focus length of the MLAs. A 31-inch MLAs with focal lengths of microlens of 9.97 mm was used in the experiment, the pitch of MLAs was calculated to be 1353 μm × 1362 μm in the horizontal and vertical directions, respectively.

The elemental image arrays were captured digitally and then displayed on the 2D curved monitor, where the depth distance between the centers of the red (front) and purple (behind) cartoon creatures was around 106.5 mm, as shown in Fig. 5c. The flexible MLAs was bent to adapt the curvature of 2D curved monitor with four stripe-like spacers sandwiched along the frame to ensure the uniformity of distance (d) and curvature. The position of MLAs was finely aligned until the sharpest reconstruction image appeared, and the assembly was then clamped and reinforced using spring clips. Figure 5d displayed the photo of curved integral imaging 3D display system with the reconstruction images, which was taken from the normal direction. It was obvious that blurry elemental image arrays were well reconstructed, showing reasonable 3D images. We also took the photos from left 45° (Fig. 5e) and right 45° (Fig. 5f) to demonstrate the 3D images from different perspectives, from which clear images could still be observed evidently. However, the floating 3D effect was not so evident, which might be due to the limited viewpoints and the low NA of the MLAs. It was concluded that, the MLAs fabricated on flexible substrates can be applied to curved integral imaging system, the viewing angle were improved obviously by using MLAs with higher NA and curved display system.

## Conclusions

In summary, large scale flexible micro-lens arrays (MLAs) were successfully fabricated on PMMA substrate based on screen printing technique. An inverted reflowing configuration allowing to take advantage of the gravity effect was implemented to improved the numerical aperture (NA) of microlenses. Furthermore, the UV resin’s viscosity and surface wettability were also optimized. The surface wettability of PMMA substrates was regulated by TMCS modification. With decreasing the substrate’s wettability, the NA values could be increased from 0.036 to 0.096, when the UV resin contact angles increased from 60.1° to 88.7°. A 31-inch flexible MLAs with 512 × 288 microlenses and the pitch of 1354 μm × 1362 μm in the horizontal and vertical directions was achieved, exhibiting good converging performance and excellent uniformity. As an example, the fabricated flexible MLAs was conbined to a curved 2D monitor to realize a curved integral imaging 3D display system, showing better 3D immersion and wider viewing angle than flat integral imaging 3D display system. The realization of curved integral imaging 3D display not only have tremendous application potentials, but also provide referential values to achieve flexible 3D displays.

## Methods

### Implementation of screen printing

**Figure 6:**
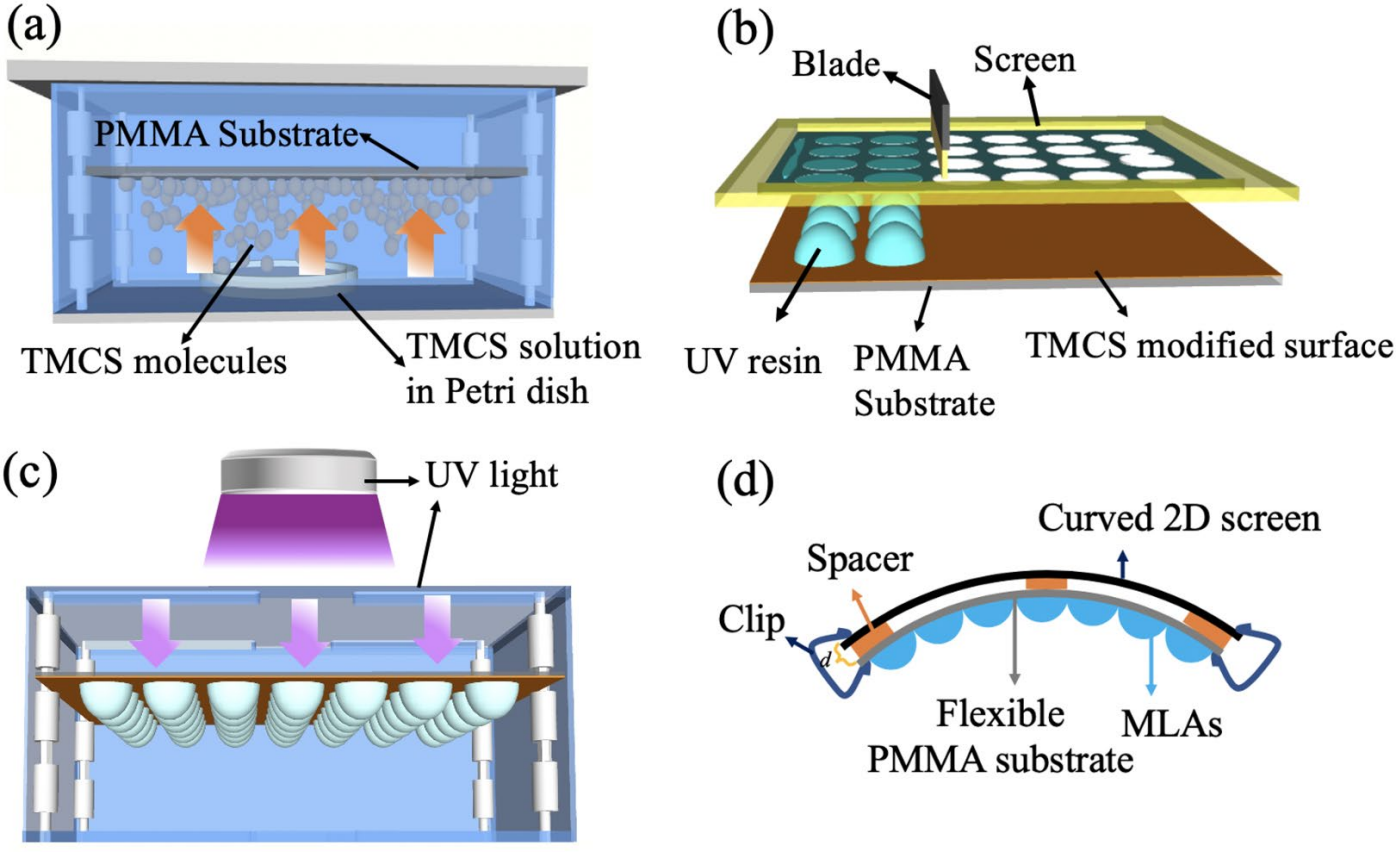
Schematic fabrication processes of MLAs. (**a**) The PMMA substrate was treated using TMCS vapor; (**b**) Schematic processes of screen printing; (**c**) The printed MLAs were laid flat with MLAs side down to reflow for various time followed by UV exposure. (**d**) Schematic of curved integral imaging 3D display system.

Flexible MLAs were fabricated using screen printing method, the detailed fabrication processes of precision composite screen and printing processes have been depicted in our previous work^21^. A series of experiments were employed to increase the NA of micro-lenses in the present work, as schematically illustrated in Fig. 6. The precision composite screen with mesh number of 325, silk diameter of 28 μm, and square opening of 50 μm were used. Flexible polymethyl methacrylate (PMMA) substrates with thickness of 2 mm were exposed to trimethylchlorosilane (TMCS, Sigma) vapor for different time to adjust the wettability of UV resin on the substrates, as shown in Fig. 6a. UV curable resin (6230G, Taiwan, China) with viscosity of around 30,000 cps was used to prepare MLAs. Another two kinds of UV resins (G1-107NX and G1-107 N, Hangzhou, China) with respective viscosities of around 50,000 cps and 4000 cps were mixed with different ratios to test the effects of viscosity on the morphology of MLAs. The UV resin was degassed in a low vacuum chamber for 1 h to remove bubbles before screen printing, employing a semi-automatic printing system (ATMACE1014, Taiwan, China). Micro-cylinder arrays (MCAs) will form by printing UV resin from the open mesh apertures on PMMA substrates, whose shapes will alter before solidification. The MCAs on PMMA substrates were laid flat with MCAs side up or MCAs side down (Fig. 6c) for various time to reflow until the spherically shaped microlenses were formed, followed by solidifying under UV light (365 nm) to form neat MLAs.

### Characterization of micro-lens arrays

Optical microscope (BX51M, OLYMPUS, Japan) and laser 3D microscope (OLS4100, OLYMPUS, Japan) were applied for the shape characterization of MLAs. Atomic force microscope (AFM, Bruker Multimode 8) in contact mode was employed to further determine the surface roughness of MLAs. The contact angles were determined using instrument (SL200KS, Kono, USA), and in all case, at least five individual measurements on different positions were taken, resulting in a mean value. The optical performances of MLAs were examined using a measurement system described in the previous report^39^. Briefly, laser beam of 650 nm was expanded and then incident on the MLAs vertically, the transmission light was then magnified and was collected by a CCD camera equipped with beam analyzer (BC106N-VIS/M, THORLABS), the pixel size of the CCD camera is 6.45 × 6.45 μm.

### Construction of curved integral imaging 3D display system

The fabricated flexible MLAs were applied to the reconstruction process of integral imaging. A curved monitor (CQ32G1, AOC) with resolution of 2560 × 1440, pixel pitch of 272.4 μm and curvature rating of 1700R (i.e. the radius of curved monitor is 1700 mm) was used. The elemental image arrays were generated using 3DS MAX and MATLAB softwares. 3D object was created by 3DS MAX modeling software and was imported in Matlab source codes. The pick-up procedure of elemental image was then picked up using simulating light field camera, which was virtually simulated using computer graphics techniques by tracing the desired rays emanating from the 3D object from different perspectives. An individual elemental image can be recorded by the sensor from a specific perspective. The individual elemental images were then put together to form elemental image arrays, which were then display on the curved monitor. The fabricated MLAs were then fixed in front of the monitor with distance of around 10 mm using clamps, depending on the parameters of MLAs. A flexible frame with thickness of around 10 mm was inserted between the monitor and the FMLAs to ensure the distance uniformity, as shown in Fig. 6d.
